# Proximity Interactome Analysis of Super Conserved Receptors Expressed in the Brain Identifies EPB41L2, SLC3A2, and LRBA as Main Partners

**DOI:** 10.3390/cells12222625

**Published:** 2023-11-14

**Authors:** Abeer Kaafarani, Romain Darche-Gabinaud, Xavier Bisteau, Virginie Imbault, Valérie Wittamer, Marc Parmentier, Isabelle Pirson

**Affiliations:** Institut de Recherche Interdisciplinaire en Biologie Humaine et Moléculaire (IRIBHM), Université Libre de Bruxelles (ULB), 1070 Brussels, Belgium; romain.darche@ulb.be (R.D.-G.); xavier.bisteau@ulb.be (X.B.); virginie.imbault@ulb.be (V.I.); valerie.wittamer@ulb.be (V.W.); marc.parmentier@ulb.be (M.P.)

**Keywords:** orphan GPCR, SREBs, Bio-ID2, proximity-dependent biotinylation, EPB41L2, SLC3A2, LRBA

## Abstract

The Super-Conserved Receptors Expressed in the Brain (SREBs) form a subfamily of orphan G protein-coupled receptors, highly conserved in evolution and characterized by a predominant expression in the brain. The signaling pathways activated by these receptors (if any) are presently unclear. Given the strong conservation of their intracellular loops, we used a BioID2 proximity-labeling assay to identify protein partners of SREBs that would interact with these conserved domains. Using streptavidin pull-down followed by mass spectrometry analysis, we identified the amino acid transporter SLC3A2, the AKAP protein LRBA, and the 4.1 protein EPB41L2 as potential interactors of these GPCRs. Using co-immunoprecipitation experiments, we confirmed the physical association of these proteins with the receptors. We then studied the functional relevance of the interaction between EPB41L2 and SREB1. Immunofluorescence microscopy revealed that SREB1 and EPB41L2 co-localize at the plasma membrane and that SREB1 is enriched in the β-catenin-positive cell membranes. siRNA knockdown experiments revealed that EPB41L2 promotes the localization of SREB1 at the plasma membrane and increases the solubilization of SREB1 when using detergents, suggesting a modification of its membrane microenvironment. Altogether, these data suggest that EPB41L2 could regulate the subcellular compartmentalization of SREBs and, as proposed for other GPCRs, could affect their stability or activation.

## 1. Introduction

G protein-coupled receptors (GPCRs) constitute the largest family of membrane receptors (~850), which are involved in the regulation of most physiological processes. GPCRs are activated by various stimuli (neurotransmitters, peptides, lipids, sugars, and light) that trigger a conformational change in the receptor and the activation of heterotrimeric G proteins, thus initiating a downstream signaling cascade [1]. Additionally, most GPCRs can induce G protein-independent signaling, following their interaction with kinases that phosphorylate the receptors, thereby promoting the recruitment of β-arrestins [2]. GPCRs and their respective ligands constitute a major class of therapeutic targets, with 34–36% of FDA-approved drugs acting through GPCRs [3]. Despite many efforts to identify their biological functions, 121 GPCRs remain as orphan receptors, as they have not yet been linked to widely accepted endogenous ligands (http://www.guidetopharmacology.org (accessed on 17 July 2023)). Understanding the physiological and pathological roles of these orphan receptors is crucial, as they could potentially serve as novel and promising therapeutic targets [4].

The Super-Conserved Receptors Expressed in the Brain (SREBs) constitute an orphan GPCR subfamily, mostly studied in mammals, and are composed of three members encoded by *SREB1* (*GPR27*), *SREB2* (*GPR85*), and *SREB3* (*GPR173*) genes [5,6,7]. These receptors exhibit high conservation across vertebrate species [6]. The amino acid sequence of GPR85/SREB2 is 100% identical between humans and mice, and it remains extremely well-conserved in various other vertebrate species, including zebrafish (89%). GPR27/SREB1 and GPR173/SREB3 are slightly more divergent, sharing respectively 97% and 98% amino acid identity between humans and mice [6]. This strong conservation across species contributes to the substantial interest in this GPCR subfamily and suggests important roles of SREBs in physiological processes.

The three receptors are prominently expressed in the central nervous system and in the pituitary gland, albeit not exclusively [6,8]. SREB1 has been proposed to play a role in neuronal cell survival, insulin synthesis and secretion [9,10], lipid metabolism [11], progression of hepatocellular carcinoma [12], and inflammatory responses linked to food allergies [13]. SREB2 function has mostly been studied in the brain, where its over-expression in mice affected behavior and decreased brain weight [14]. Conversely, mice lacking SREB2 exhibited an opposite phenotype [15]. Genetic variants of SREB2 were also described as risk factors for schizophrenia [16] and autism spectrum disorder [17]. SREB3 was proposed to function as a receptor for the pentapeptide gonadotropin-releasing hormone (GnRH-(1–5)), as well as for a novel reproductive peptide known as phoenixin-20 amide (PNX). In this context, SREB3 was proposed to inhibit the migration of GnRH neurons, to control the activity of the hypothalamus [18,19,20], and to regulate the reproductive system by impacting the estrous cycle length [21]. Recently, two chemical compounds were described as selective agonists of a chimeric form of SREB1, promoting β-arrestin recruitment [22] and the chemokine CXCL14 as an endogenous ligand of SREB2 [23]. Nevertheless, despite these synthetic and endogenous ligands proposed for the three SREBs, these receptors are still considered as orphans by the International Union of Basic and Clinical Pharmacology (IUPHAR).

SREBs are classified in the rhodopsin-like family (class A) of GPCRs, with some similarity with prostaglandin, purinergic, and amine receptor groups [6,24,25]. However, most of the conserved molecular elements of the rhodopsin-like receptors family playing key roles in the activation process [26] are not present or differ in SREB sequences [27]. For instance, the DRY motif at the junction of transmembrane domain (TM) III and intracellular loop (ICL) 2 is replaced by a TRY motif, the NPxxY motif in TM VII is replaced by NPxxC, and leucine replaces the cysteine in the CWxP motif in TM VI. In addition, there are no prolines in TM V [6]. The absence of these typical features of rhodopsin-like GPCRs raises many questions regarding the function of SREBs. They may be atypical GPCRs devoid of signaling properties or signaling though unconventional pathways. They may also have protein interaction partners differing from those known for other rhodopsin-like GPCRs or lack endogenous ligands entirely [27]. Mutations in the DRY motif are known to impair the stability of receptors in their inactive state, unleashing constitutive activity [27,28,29]. In this regard, constitutive activity was only proposed for SREB1. Over-expression of SREB1 in HEK293T cells was reported to increase inositol phosphate levels, while cAMP levels were not significantly changed, suggesting that SREB1 is coupled to Gq/11 [10]. However, this was not confirmed thereafter.

The SREB share 52 to 63% amino acid identity between them. Generally, transmembrane segments are the most conserved domains between GPCR orthologs and within GPCR families [28]. These regions are strongly conserved for each SREB across species but are more variable between the three SREB members. The extracellular domains and the intracellular C-terminus appear as the most variable parts amongst SREB members. On the contrary, the three intracellular loops, particularly the long third loop, display a remarkable conservation between the three SREBs. Approximately, 60% of the amino acids of this third loop are identical between the three SREB sequences from humans, mice, and zebrafish. This very strong conservation, which is unusual for GPCRs, may indicate a functional specificity of this group of receptors and suggest potential interactions with common intracellular partners. Identifying proteins interacting with SREBs could help to elucidate the intracellular environment and signaling pathways of these receptors. To this end, we performed a proximity-labeling assay using BioID2 as biotin ligase coupled to affinity purification and mass spectrometry [29,30,31] and identified new interactors of SREBs, including EPB41L2, SLC3A2, and LRBA. We analyzed, in more detail, the functional impact of EPB41L2 expression on SREB1 and showed that it influences the solubilization and the subcellular localization of the receptor.

## 2. Materials and Methods

### 2.1. Antibodies

The primary and secondary antibodies used in this work are the following: mouse anti-BioID2 (#ab2232733, Abcam, Cambridge, UK, WB/IF 1:1000), rabbit anti-HA (#3724, Cell Signaling, Waltham, MA, USA, WB 1:1000), mouse anti-HA antibody (#MMS101R, Covance, Princeton, NJ, USA, IP 1:100), rabbit anti-myc (#2278, Cell Signaling, Waltham, MA, USA, WB 1:1000, IF 1:400), rabbit anti-FLAG (#PA1-984B, ThermoFisher, Waltham, MA, USA, WB 1:1000), mouse anti-GAPDH (#MAB374, Chemicon, Tokyo, Japan, WB 1:30,000), rabbit anti-EPB41L2 (#ab175928, Abcam, Cambridge, UK, WB 1:2000, IF 1:300), rabbit anti-β catenin (#8814, Cell Signaling, Waltham, MA, USA, 1:300), horseradish peroxidase (HRP)-conjugated goat anti-mouse IgG (#31430, Invitrogen, Waltham, MA, USA, 1:50,000), HRP-conjugated goat anti-rabbit IgG (#31460, Invitrogen, Waltham, MA, USA, 1:50,000), Alexa-Fluor-647-conjugated goat anti-mouse IgG (#A-32728, Invitrogen, Waltham, MA, USA, 1:500) and Alexa-Fluor-488-conjugated donkey anti-rabbit IgG (#A-21206, Invitrogen, Waltham, MA, USA, 1:500). The HRP-conjugated streptavidin was #ab7403 ( Abcam, Cambridge, UK, WB 1:4000).

### 2.2. DNA Constructs

**Figure 1:**
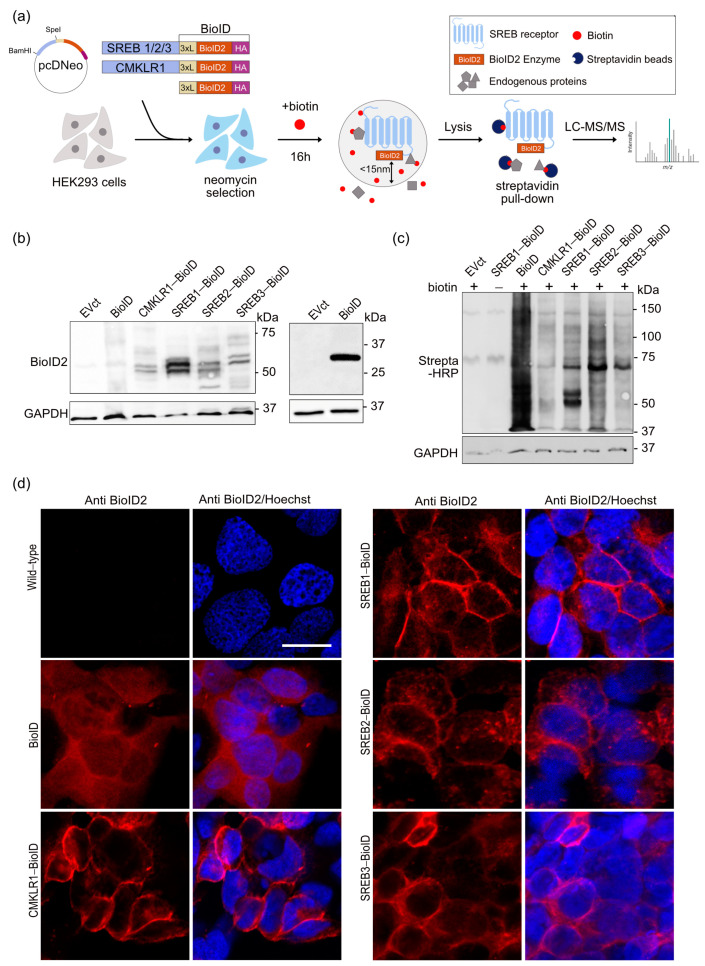
SREB-BioID fusion proteins are expressed, functional in biotinylation, and localized at the plasma membrane. (**a**) Experimental workflow of BioID2 proximity-based labeling. In the pcDNeo vector, sequences encoding a 3×(GGGGS)-linker (3×L), BioID2, and an influenza hemagglutinin (HA) tag (BioID) were fused to the C-terminus of the receptors. BioID alone was used as the control. Stable HEK293 cell lines expressing these constructs were selected using G418 and labeled using biotin for 16 h. BioID2 biotinylates proteins had a radius of about 15 nm. Lysates were pulled-down using streptavidin beads and analyzed using LC-MS/MS to identify interacting proteins. (**b**) Total lysates of HEK293 cells transiently expressing the indicated constructs were analyzed via Western blotting (following 8% polyacrylamide gel (left panel) or 12% (right panel)) and the fusion proteins were detected using an anti-BioID2 antibody. GAPDH was used as a loading control. (**c**) After 16 h of treatment, the efficiency of biotinylation was assessed using HRP-conjugated streptavidin. The Western blotting images are representative of three independent experiments. (**d**) In cells stably expressing BioID, CMKLR1, or SREB1/2/3 fusions, the distribution of proteins was monitored using immunofluorescence. Cells expressing the constructs were mixed with control (Ctrl) HEK293 cells and stained using anti-BioID2 antibodies (red) and Hoechst (blue). Images were obtained using a confocal inverted Zeiss LSM 780 microscope and a 40× objective. Scale bars: 20 µm.

A construct encoding a fusion of human SREB1 (GPR27, Gene ID: 2850, http://www.ncbi.nlm.nih.gov/genbank (accessed on 20 July 2023)), a 3×(GGGGS)-linker, the second-generation biotin ligase derived from Aquifex aeolicus (BioID2), and an influenza hemagglutinin (HA) tag was custom synthesized using GenScript and inserted into a homemade bicistronic vector (pcDNeo). The linker-BioID2-HA moiety will be referred to as BioID in this paper. DNA fragments encoding human SREB2 (GPR85, Gene ID: 54329), SREB3 (GPR173, Gene ID: 54328), and CMKLR1 (Gene ID: 1240) were amplified using PCR and subcloned in frame between the *Bam*HI and *Spe*I sites of the pcDNeo-BioID vector, after removal of the SREB1 sequence (Figure 1). A BioID-alone fragment was also amplified and cloned between the *Bam*H1 and *Not*I sites of pcDNeo. The following primers were used for amplification (restriction sites are in bold and underlined):

(SREB2-For): 5′-CG**G**GATCCACCATGGCGAACTATAGCCATGCA-3′;

(SREB2-Rev): 5′-CGACTAGT TATAACACAGTAAGGTTCCCTTGGTA-3′;

(SREB3-For): 5′-CGGGATCCACCATGGCCAACACTACCGGAGAG-3′;

(SREB3-Rev): 5′-CGACTAGT CATGACACAGTAGGGTTCTCTGGGAG-3′;

(CMKLR1-For): 5′-CGGGATCCACCATGGAGGATGAAGATTACAAC-3′;

(CMKLR1-Rev): 5′-CGACTAGTAAGCATGCCGGTCTCCCTCTCATTC-3′;

(BioID-alone-For): 5′-CGGGATCCACCATGGGTGGAGGCGGGTCTGGAGGC-3′;

(BioID-alone-Rev): 5′-CTAGGCGGCCGCCTATGCGTAATCCGGTACATC-3′.

The PCR conditions were 95 °C for 3 min; 95 °C for 30 s, 58 °C for 1 min, and 72 °C for 90 s, 30 cycles; and 72 °C for 10 min, using Pfu-Turbo DNA polymerase (Invitrogen, Waltham, MA, USA). All plasmids were verified via sequencing. DNA fragments encoding EPB41L2 (Gene ID: 2037), EPB41L3 (Gene ID: 23136), SLC3A2 (Gene ID: 17254), LRBA domain 1 (1–747 aa), LRBA domain 2 (748–1864 aa), LRBA domain 3 (1865–2576 aa), and LRBA domain 4 (2577–2863 aa) were custom synthesized using GeneCust (Boynes, France) and cloned in pcDNA3.1(+) myc-His A. The SREB1 coding sequence was cloned in pcDNA-FLAG and used for solubilization experiments.

### 2.3. Cell Culture and Transfections

Human embryonic kidney 293 (HEK293) or HEK293T cell lines (American Type Culture Collection (ATCC), CRL-3216, and CRL-1573) were used for stable and transient expression, respectively. Cells were cultured in Dulbecco’s modified Eagle’s medium (DMEM) supplemented with 10% fetal bovine serum, 50 units/mL penicillin, 50 µg/mL streptomycin, 2.5 µg/mL fungizone, and 1 mM sodium pyruvate. All components were obtained from ThermoFisher Scientific (Waltham, MA, USA). Cells were seeded in 6-well plates (3 × 10^5^ cells/well) and transfected with 2 µg (for stable expression) or 3 µg (for transient expression) of the different plasmids using X-tremeGENE^TM^ 9 DNA Transfection Reagent (Roche Life Science, Basel, Switzerland) as recommended by the manufacturer, with a ratio of 3 µL of reagent for 1µg of plasmid DNA. Selection of stably transfected cells was carried out in culture medium supplemented with 0.5 mg/mL G418 (#108321-42-2, InvivoGen, San Diego, CA, USA). After 10 to 15 days of selection, individual colonies were transferred to 24-well plates, grown to confluency, trypsinized, expanded further in 6-well plates, and validated via a reverse transcription polymerase chain reaction (RT-PCR).

### 2.4. RNA Extraction and qPCR

To validate the expression of fusion constructs in clones, total RNA was extracted using an optimized procedure combining solubilization in TRIzol^TM^ reagent (Invitrogen, Waltham, MA, USA) followed by purification using the RNeasy Mini Kit (Qiagen, Hilden, Germany). Genomic DNA was removed using the RNase-Free DNase Set (Qiagen, Hilden, Germany). cDNA was synthesized using the SuperScript II Reverse Transcriptase and Oligo(dT) (ThermoFisher Scientific, Waltham, MA, USA). All procedures were performed under RNAse-free (RF) conditions, following the manufacturer’s recommendations. RT-PCR was performed using a touch down program (94 °C for 2 min 30 s; 94 °C for 30 s, 65 °C for 30 s (with a decrease of 0.5 °C per cycle), 72°C for 45 s, 20 cycles; 94 °C for 30 s, 55 °C for 30 s, 72 °C for 45 s, 30 cycles; and 72 °C for 10 min) using the primer pairs detailed in Supplementary Table S1.

### 2.5. BioID Assay

BioID pull-down experiments were conducted according to Roux et al. protocol [29]. Briefly, for each experimental condition, four 10 cm dishes at 80% confluency were incubated with 50 µM biotin (Sigma-Aldrich, St. Louis, MO, USA) in complete medium for 24 h. The cells were then rinsed twice with cold PBS then lysed on ice with 50 mM Tris-HCl, pH 7.4, containing 8 M urea, 1 mM DTT and 1 × cOmplete™ Protease Inhibitor Cocktail (Roche, Basel, Switzerland), and harvested. A total of 1% Triton X-100 was added, and two sonication sessions of 15 pulses were applied using a Vibra-Cell device (Sonics, Oklahoma City, OK, USA) at an amplitude of 30% and using a 1 s on/2 s off program. The cell lysates were then centrifuged at 15,000× *g* for 10 min at 4 °C. Each experimental condition was divided into 3 technical replicates. The cleared supernatant (1 mg of total proteins) was added to pre-equilibrated Streptavidin Sepharose High Performance Beads (GE Healthcare, Chicago, IL, USA) and incubated overnight at 4 °C with constant rotation. The beads were then harvested and washed 4 times with 50 mM Tris-HCl, pH 7.4, containing 8 M urea for 8 min with rotation followed by successive centrifugations. A total of 1 mM biotin in 50 mM ammonium bicarbonate was then added to the beads to bind all remaining unbound streptavidin and stabilize the proteins. Finally, the beads were rinsed with PBS and processed for mass spectrometry analysis.

### 2.6. Mass Spectrometry

Dried agarose beads were resuspended in 100 mM Tris-HCl pH 8.5 containing 1% sodium deoxycholate, 10 mM tris(2-carboxyethyl)phosphine (TCEP), and 55 mM chloroacetamide (SDC buffer), and denatured for 10 min at 95 °C. Samples were diluted two-fold with 100 mM triethylammonium bicarbonate buffer (TEAB) and proteins were digested during 3 h with 1 µg of trypsin (Promega #V5111, Madison, WI, USA) and 1 µg of LysC (Wako #129-02541, Monza, Italy) at 37 °C. Peptides were purified using SDB-RPS columns (Affinisep, Le Houlme, France). Briefly, digested peptides were diluted two-fold with 2% TFA in isopropanol, mixed thoroughly, and loaded on an SDB-RPS column. After washing with 1% TFA in isopropanol followed by 5% ACN/0.2% TFA, peptides were eluted with 5% NH_4_OH/60% ACN and evaporated to dryness at 45 °C. A total of 80% of the resuspended peptides (8/10 µL in 100% H_2_O/0.1% HCOOH) were injected on a TripleTOF 5600 mass spectrometer (Sciex, Concord, NSW, Canada), interfaced to an EK425 HPLC System (Eksigent, Dublin, CA, USA), and data were acquired using a Data-Dependent-Acquisition (DDA) mode. Peptides were injected on a separation column (Eksigent ChromXP C18, 150 mm, 3 µm, 120 Å) using a two-step acetonitrile gradient (5–25% can/0.1% HCOOH in 48 min then 25–60% ACN/0.1% HCOOH in 20 min at 5 µL/min) and were sprayed on-line in the mass spectrometer. MS1 spectra were collected in the range 400–1250 *m*/*z* with an accumulation of 250 ms. The 20 most intense precursors with a charge state of 2–4 were selected for fragmentation, and MS2 spectra were collected in the range of 50–2000 *m*/*z* with an accumulation of 100 ms; precursor ions were excluded for reselection for 12 s.

### 2.7. Data Analysis

Raw data were analyzed using the Fragpipe computational platform (v19.1) with MSfragger [32] (v3.7), Philosopher [33] (v4.8.1), and IonQuant [34] tools. Peptide identifications were obtained by using the MSFragger search engine on .mzML files, from converted .Wiff/Wiff.scan files, on a human protein sequence database (UP000005640) from Uniprot (downloaded 25 January 2023; contained “sp” and “tr” sequences; no isoforms) supplemented with common contaminant proteins and reversed protein sequences as decoys. Mass tolerances for precursors and fragments were set to 30 and 20 ppm, respectively, with spectrum deisotoping mass calibration [35] and parameter optimization enabled. Enzyme specificity was set to “trypsin” with enzymatic cleavage and a maximum of 5 missed trypsin cleavages was allowed. Isotope error was set to 0/1/2. Peptide length was set from 6 to 50, and peptide mass was set from 500 to 5000 Da. Variable modifications (methionine oxidation, acetylation of protein N-termini, deamidation of Q and N [+0.984016Da], biotin addition onto lysine [+226.0776], pyro-Glu/Gln [−18.0106/−17.0265 Da], and carbamylation of lysine and protein terminus [+43.005814]) were added, while carbamidomethylation of Cysteine was set as a fixed modification. The maximum number of variable modifications per peptide was set to 5. MS/MS search results were further processed using the Philosopher toolkit with PeptideProphet (with options for accurate mass model binning: semi-parametric modeling with computation of possible non-zero probabilities for decoy entries) and with ProteinProphet. Further filtering to 1% protein-level FDR allowing unique and razor peptides was used and final generated reports were filtered at each level (PSM, ion, peptide, and protein) at 1% FDR. Label-free quantification was performed using IonQuant with normalization enabled, a 4 ions minimum, and default advanced options. Further statistical analysis and visualization were performed in R (v4.1) using commonly used packages. SAINT [36,37] scoring and fold change (FC-B using geometric mean) was performed using the total spectral count without prior normalization and using defaults parameters on the REPRINT webtools [38] and using our provided control samples for the analysis.

### 2.8. Western Blotting, Co-Immunoprecipitation, and Antibodies

For Western blotting analysis, the culture medium was removed and the cells were washed in cold phosphate-buffered saline (PBS) and lysed in 50 mM Tris-HCl, pH 6.8, containing 12% sucrose, 2% SDS, 0.004% bromophenol blue, 20 mM dithiothreitol (DTT), and 1 × cOmplete™ Protease Inhibitor Cocktail (Roche, Basel, Switzerland). The lysates were cleared via centrifugation at 15,000× *g* at 4 °C and the total protein concentration was measured using the PierceR 660 nm Protein assay reagent (22660, Thermo Scientific, Waltham, MA, USA). The samples of total cell extracts (30 µg of proteins) were denatured at 65 °C for 15 min in Laemmli Buffer 1 × (65 mM Tris-HCl pH 6.8, 2% SDS, 5% glycerol, 0.01% bromophenol blue, and 0.1% DTT). Proteins were separated using SDS-PAGE, followed by Western blotting, and revealed via enhanced chemiluminescence (ECL) using the Western Lightning Plus system (PerkinElmer, Waltham, MA, USA). For immunoprecipitations, cells were lysed in 50 mM Tris-HCl, pH 8.0, containing 1% NP40, 0.5% deoxycholate, 0.1% SDS, 150 mM NaCl, 5 mM EDTA, 10 mM Na_4_P_2_O_7_, and 1 × cOmplete™ Protease Inhibitor Cocktail (Roche, Basel, Switzerland). The lysate (1 mg of total proteins) was incubated with G-Sepharose beads (GE healthcare, Chicago, IL, USA) pre-coupled to mouse anti-HA antibody 1:100 (Covance MMS101R) at 4 °C for 4 h with constant rotation. The beads were then washed twice with lysis buffer without inhibitors and twice with lysis buffer without inhibitors or detergents. After the last centrifugation, the complexes were dissolved in 2 × Laemmli buffer, denatured at 65 °C for 15 min, loaded on an SDS-PAGE acrylamide gel, and analyzed via Western blotting. To test immunoprecipitation in the presence of 2,4-dichloro-N-{4-[(1,3-thiazol-2-ylamino)sulfonyl]phenyl}benzamide (ID5217941, ChemBridge, San Diego, CA, USA), cells were treated with 15 µM of the ID5217941 compound for 15 min before lysis and the compound was maintained in the lysis and washing buffers.

### 2.9. siRNA Knock-Down

A stable HEK293 cell line expressing SREB1-BioID was transfected with 100 nM of a SMART pool of human EPBL1L2 siRNA (L-003661-02-0005) or a non-target control siRNA (#D-001810-10-20) with DharmaFECT 1 transfection reagent according to manufacturer’s protocol. All materials were purchased from Dharmacon (Lafayette, CO, USA). A total of 48 h after transfection, the cells were lysed in 50 mM Tris-Cl pH 8 containing 1% NP40, 0.5% deoxycholate, 0.1% SDS, 150 mM NaCl, 5 mM EDTA, 10 mM Na_4_P_2_O_7_, and 1 × cOmplete™ Protease Inhibitor Cocktail (Roche, Basel, Switzerland) for Western blotting or fixed with 4% PAF for immunofluorescence experiments.

### 2.10. Immunofluorescence

Cells were plated on coverslips (Ø 12 mm and #1.5H) (Thermo Fisher Scientific, Waltham, MA, USA) precoated with 50 μg/mL poly D-lysine (Gibco, Waltham, MA, USA), washed twice with PBS, fixed with 4% paraformaldehyde (PFA) for 15 min at room temperature, and blocked and permeabilized for 1 h at room temperature in PBS containing 0.1% Triton X-100 (PBS-TX) and 10% horse serum (HS). The coverslips were incubated overnight at 4 °C with the primary antibodies, in PBS-TX containing 1% HS. They were rinsed in PBS-TX and incubated with the secondary antibodies in PBS-TX containing 1% HS in the dark. Coverslips were then rinsed in PBS-TX. The nuclei were stained for 2 min at room temperature with Hoechst (1/2000) in PBS. After two washes in PBS, the coverslips were mounted with FluoroSave reagent (Merck, Rahway, NJ, USA). The images were obtained with a confocal inverted Zeiss LSM 780 microscope (63× objective), using 405 nm excitation for Hoechst, 488 nm for Alexa Fluor, and 488 and 633 nm for Alexa Fluor 647. For quantification of proteins at the plasma membrane, fluorescence profiles were plotted using RGB graphs on Image J. The green fluorescent regions of interest (ROI) were selected in cells chosen at random; the intensity of far-red fluorescence was quantified in the same region. For quantification of the knockdown efficiency, the fluorescence intensity of EPB41L2 in the ROI was measured using Image J and normalized to the area.

### 2.11. Solubilization Experiments

Cells co-transfected with plasmids encoding the receptor and the potential interactor were lysed in 50 mM HEPES, pH 7.5, containing 0.5% n-dodecyl-b-maltoside (DDM), 150 mM NaCl, 10 mM EDTA, 2 mM orthovanadate, 10 mM NaF, 10 mM Na_4_P_2_O_7_, and 1 × cOmplete™ Protease Inhibitor Cocktail (Roche, Basel, Switzerland). After centrifugation, the supernatant was collected and the pellet was solubilized in 1 × Laemmli buffer, sonicated for one session of 10 pulses using a Vibra Cell ultrasonic processor (Sonics) at an amplitude of 30% using a 1 s on/2 s off program, and then centrifuged at 15,000× *g* for 15 min at 4 °C. For total protein extraction, cells were lysed in 1 × Laemmli Buffer 65 mM Tris-HCl, pH 6.8, containing 2% SDS, 5% glycerol, 0.01% bromophenol blue, 0.1% DTT, and 1 × cOmplete™ Protease Inhibitor Cocktail (Roche, Basel, Switzerland).

### 2.12. Quantification and Statistic Analysis

Quantification of Western blot signals was conducted using ImageJ software (version 1.54d, National Institute of Health, Bethesda, MD, USA). The GraphPad Prism software (version 6.01, Dotmatics, Boston, MA, USA) was used for statistical analyses using the *t*-test (* *p* ≤ 0.05; ** *p* ≤ 0.01; and *** *p* ≤ 0.001) and Pearson’s correlation coefficient

## 3. Results

### 3.1. BioID2 Fused to SREB Family Members Is Functional and the Fusion Proteins Are Expressed and Localize at the Plasma Membrane

Aiming to identify proximal interactors of SREB receptors, we performed a promiscuous biotinylation assay using BioID2 as biotin-ligase [30]. First, we cloned the receptors in fusion with a 3 × (GGGGC)-linker sequence, forming a flexible arm, followed by the BioID2 sequence C-terminally tagged with HA (Figure 1a). The linker-BioID2-HA sequence will, hereafter, be referred to as BioID. CMKLR1 (chemerin receptor) was used as an unrelated GPCR control. We first sought to assess the transient expression of the fusion proteins in HEK293 cells transfected with the constructs. GPCR-BioID expression was monitored via Western blotting using anti-BioID2 (Figure 1b) or anti-HA (Figure S1) antibodies. We confirmed the equal protein load via Ponceau staining of the membranes (Figure S1) and via immunodetection of the housekeeping protein glyceraldehyde 3-phosphate dehydrogenase (GAPDH) (Figure 1b). As the expression in mammalian cell lines produces recombinant receptors with post-translational modifications (PTMs), multiple sets of bands were observed for each GPCR. The same pattern of bands was observed with both antibodies (anti-HA and anti-BioID2), confirming that our constructs were well expressed and not significantly degraded.

Cells were then incubated for 16 h in the presence of 50 µM biotin and lysed. The protein extracts were analyzed via Western blotting using HRP-streptavidin to evaluate the efficiency of the biotinylation process. This revealed biotinylation of many proteins in the conditions expressing the GPCR-BioID or BioID alone compared to cells transfected with an empty vector or transfected with SREB1-BioID but without incubation with biotin (Figure 1c). Different profiles of labeled proteins were observed in the five experimental conditions, suggesting that the five fusion proteins labeled endogenous proteins differentially (Figure 1c).

We then generated HEK293 cell lines stably expressing BioID-tagged SREB1, SREB2, SREB3, CMKLR1, and BioID alone to assess the subcellular localization of the fusion proteins. After 10–12 days of selection with G418, the clones were purified and transcripts encoding the fusion proteins were detected via RT-PCR (Figure S2). The selected clones were co-cultured with control wt HEK293 cells. Via immunofluorescence microscopy, we confirmed, using anti-BioID2 antibodies, that the receptors fused with BioID were expressed and that they were detected at the plasma membrane. On the contrary, BioID protein alone was detected to be diffused in the cytoplasm of the cells. No signal was observed in control HEK293 cells (Figure 1d).

### 3.2. SREBs Interactome-Mapped using Proximity-Based Biotinylation

**Figure 2:**
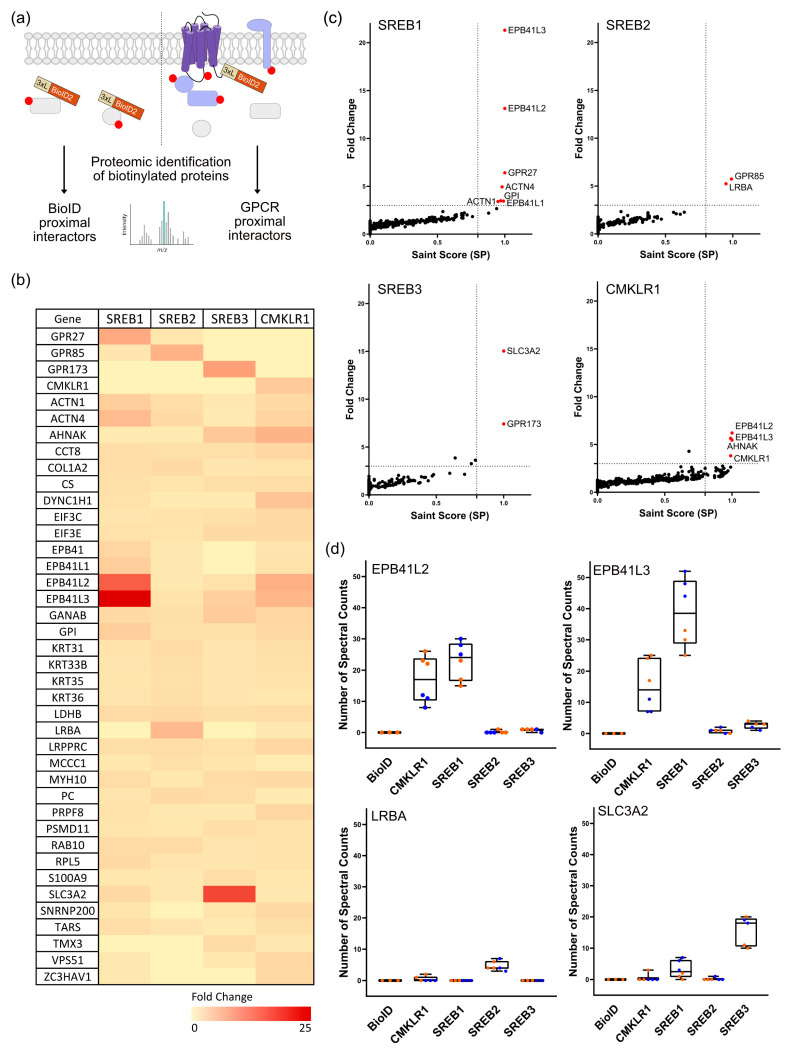
Proximal interactors of SREBs identified in BioID assays: (**a**) Schematic overview of the proteomic identification of biotinylated proteins in BioID assay. (**b**) Heatmap of the 40 most enriched proteins for the BioID baits of SREB1, SREB2, SREB3, and CMKLR1. The color code provides the fold change of the number of spectral counts of each candidate identified for the receptors. (**c**) Scattered plots of the identified proteins for each BioID-fused receptor. Red-labeled dots indicate the proximal interactors presenting a spectral counts fold change equal to or higher than three and a SAINT score (SP) equal to or above 0.8. (**d**) Reproducibility boxplot of the number of spectral counts quantified in each biological and technical replicate of each sample for EPB41L3, EPB41L2, LRBA, and SLC3A2enriched proteins. (*n* = 2 independent experiments; orange: biological clone A; blue: biological clone B).

Having established proper expression, localization, and BioID2 functionality for all constructs, we performed pull-down experiments. Clones stably expressing the different constructs were exposed to biotin and total protein extracts were prepared. Biotinylated proteins were isolated using streptavidin-affinity purification (AP) and analyzed using LC–MS/MS (Figure 2a). Two independent experiments, using two different clones, were performed for each construct, allowing the identification of a total of 1030 proteins in all combined pull-down experiments. As a quantitative measure to represent the relative abundance of proteins in the biological sample, we used spectral counts (spc), which indicate the number of mass spectra assigned to precursor peptides derived from a specific protein. For all biological and experimental replicates, the correlation between samples was good with mean Pearson coefficient (R) of 0.87 for technical replicates and 0.8 for biological replicates, indicating the good reproducibility of the procedure (Figure S3).

This approach identified various biotinylated proteins specific for each bait, as well as carboxylase enzymes, pyruvate carboxylase (PC), propionyl-CoA carboxylase alpha chain (PCCA), acetyl-CoA carboxylase 1 (ACACA), and methylcrotonoyl-CoA carboxylase subunit alpha (MCCCA) in all samples, which are naturally biotinylated in human cells, thereby confirming a successful capture.

The enrichment of individual proteins was determined using the stringent empirical fold-change (FC-B) calculation using the geometric mean of spectral counts of the top 3 replicates for each bait in each GPCR-BioID sample compared to the BioID control (Figure 2b). SAINT (significance analysis of interactome) scores were calculated for each bait and plotted against their fold changes. The highly enriched candidates were filtered according to inclusion criteria of a three-fold cut-off and a SAINT score (SP) of 0.8 (Figure 2c). Among the highly enriched proteins identified for each bait was the receptor itself, labelled by the BioID2 as an internal positive control: GPR27 (FC 6.4; SP: 1), GPR85 (FC 5.7; SP: 0.99), GPR173 (FC 7.4; SP: 1), and CMKLR1 (FC 3.8; SP: 0.99). This indicated that the procedure was efficient and specific (Figure 2c).

Most of the proximal interactors enriched in the three SREBs pull-down were shared with the ones identified for CMKLR1 (Figure 2b,c). The most enriched candidates for each SREB were selected for further analysis. Interestingly, we identified members of the family of erythrocyte membrane protein band 4.1 (EPB) known to be involved in maintaining the structural integrity of cell membranes [39]. Two members of this family (EPB41L2 and EPB41L3) were especially enriched in SREB1 and CMKLR1 (Figure 2d, panel 1 and 2, respectively). The lipopolysaccharide-responsive and beige-like anchor protein (LRBA), a scaffold protein involved in vesicular trafficking, autophagy, and immune cell signaling [40], was one of the significant neighbors identified for SREB2 (Figure 2d, panel 3). We also specifically identified the solute carrier family 3 member 2 protein (SLC3A2), belonging to the protein family of the solute carrier (SLC), a large group of membrane transport proteins enriched in SREB1 and SREB3 (Figure 2d, panel 4). SLC3A2 is a transmembrane glycoprotein that serves as the heavy chain subunit of L amino acid transporters [41]. Most selected candidates were not exclusive to a single receptor but identified as proximal interactors for the other SREBs and/or CMKLR1 with a lower fold change or significance. Multiple subunits of the guanine nucleotide-binding proteins (G proteins), including G_αs_, G_αi2_, and G_α11_, were identified as partners of CMKLR1 only but at low spectral counts.

### 3.3. Confirmation of the Physical Association of SREBs with EPB41L2, SLC3A2, and LRBA via Co-Immunoprecipitation

**Figure 3:**
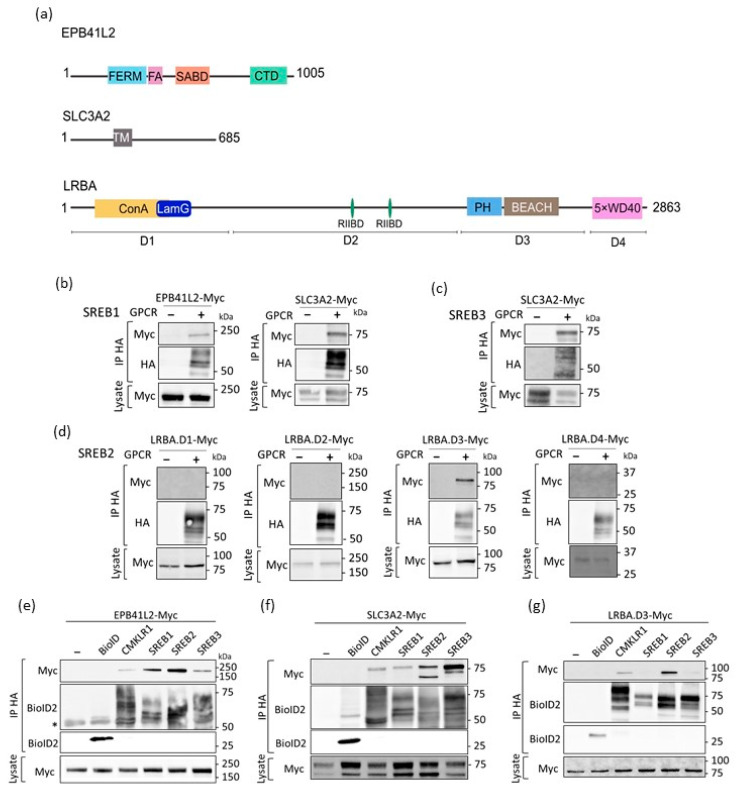
The physical interaction between the SREBs and their partners was confirmed via co-immunoprecipitation. (**a**) Schematic representation of the candidate interactors studied and their protein–protein interaction domains. HEK293T cells were transfected with SREB1 (**b**), SREB3 (**c**), or SREB2 BioID2-HA (**d**) together with Myc-tagged versions of the proximal interactors identified in the BioID assay. HEK293T cells were transfected with empty plasmid, BioID alone, CMKLR1, and SREBs together with Myc-tagged versions of the proximal interactors EPB41L2 (**e**), SLC3A2 (**f**), and LRBA.D3 (**g**). Immunoglobulin heavy chain are marked with an asterisk (*). Expression of the partners was monitored using immunoblotting of the total cell lysate. Anti-HA immunoprecipitates were analyzed using Western blotting with the indicated antibodies. Images are representative of at least three independent experiments.

We then tested whether proteins identified in this BioID screening could be confirmed as interactors via co-immunoprecipitation (co-IP) experiments. GPCR-BioID constructs were co-expressed with constructs expressing the full-length EPB41L2, EPB41L3, or SLC3A2, C-terminally tagged with a Myc epitope. LRBA, which is a large protein (316 KDa), was divided into four domains, all myc-tagged at their C-terminus (Figure 3a). The receptors were immunoprecipitated using anti-HA antibodies and the presence of the targets in the precipitates was visualized using anti-Myc immunoblotting. EPB41L2 and SLC3A2 were specifically and efficiently co-immunoprecipitated with SREB1 (Figure 3b). The interaction between EPB41L2 and SREB1 was tested in the presence of 15 µM of a proposed agonist of SREB1, the ID5217941 compound [22]. The level of EPB41L2 normalized to the level of precipitated SREB1 was measured. No difference in the amount of immunoprecipitated EPB41L2 was detected, indicating the absence of effect of ID5217941 on the interaction between SREB1 and EPB41L2 (Figure S4a). EPB41L3 (Figure S4c) and SLC3A2 (Figure 3c) were confirmed to interact with SREB3. We failed to validate the physical interaction between EPB41L3 and SREB1, due to the unspecific precipitation of the target in an anti-HA pull-down in the absence of a receptor (Figure S4b). Only LRBA domain 3 co-immunoprecipitated with SREB2, indicating that this domain could be responsible for the interaction (Figure 3d).

We also performed interaction tests for each candidate with all SREBs, CMKLR1, and BioID alone (Figure 3e–g). Given that some of the selected interactors were detected at lower enrichment levels in the other SREBs or CMKLR1 pull-down, it was not surprising to detect EPB41L2 and SLC3A2 immunoprecipitated with all three SREBs and CMKLR1. No interaction was observed with BioID alone, demonstrating the specificity of the association with the candidates (Figure 3e–g). It should be noted that SLC3A2 was observed in the extracts as two major bands, suggesting different glycosylated isoforms as a result of post-translational modifications of the protein [42]. Interestingly, SREB2 immunoprecipitated both isoforms of SLC3A2 (Figure 3f), while SREB1, SREB3, and CMKLR1 showed a physical interaction with only one of the isoforms (Figure 3f). In addition to SREB2, CMKLR1 also immunoprecipitated LRBA domain 3, suggesting the existence of a specific interaction site of LRBA with different GPCRs (Figure 3g).

### 3.4. EPB41L2 Modifies the Solubilization of SREB1 When Using Detergents

**Figure 4:**
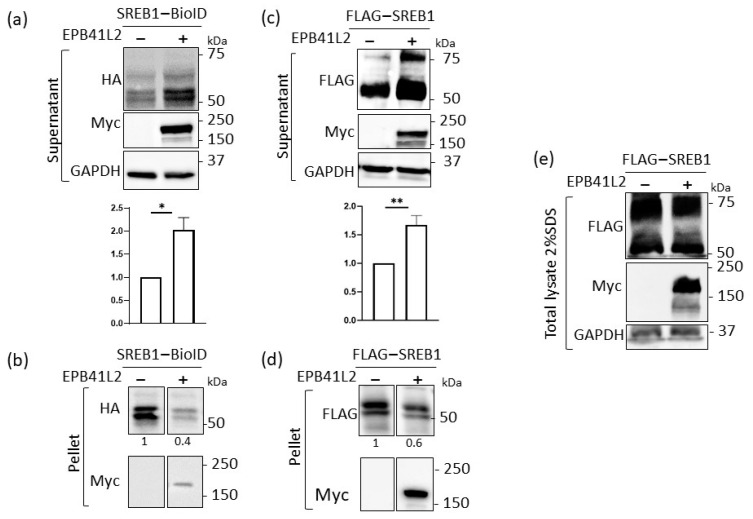
SREB1 solubilization increased upon EPB41L2 co-expression. HEK293T cells were transfected with SREB1-BioID with or without EPB41L2-Myc (**a**,**b**) or with FLAG-SREB1 with or without EPB41L2-Myc (**c**,**d**). The cells were lysed in buffer containing 0.5% DDM (a mild detergent) (**a**–**d**); SREB1 was detected in supernatants (**a**,**c**) or pellets (**b**,**d**) using an anti-HA antibody. The graphs represent the quantification (via ImageJ) of SREB1 in the soluble fraction, normalized for the amount obtained in the absence of EPB41L2. The results represent means ± SD of at least five independent experiments. Statistical significance was determined via the Student’s *t*-test (* *p* ≤ 0.05; ** *p* ≤ 0.01). (**e**) The same cells were lysed with Laemmli buffer containing 2% SDS and the lysate was analyzed, showing that the total amount of SREB1 was not modified by the co-expression of EPB41L2.

While analyzing the lysates prepared for co-IP experiments, we noted that the levels of SREB1 in the lysates were higher when EPB41L2 was co-expressed. To confirm this observation, we transiently co-expressed SREB1-BioID and EPB41L2-Myc in HEK293T cells. A total of 48 h after transfection, the cells were lysed in a buffer containing 0.5% n-dodecyl-b-maltoside (DDM), and the lysates separated into soluble (supernatant) and non-soluble (pellet) fractions. Anti-HA detection showed an increased level of SREB1 in the soluble fraction (Figure 4a) and a lower amount in the pellet (Figure 4b) upon co-expression of EPB41L2. We confirmed equal load via GAPDH detection. To verify that this effect was not due to the presence of the BioID moiety at the C-terminus of SREB1, we used an N-terminally FLAG-tagged SREB1 construct and made the same observation after anti-FLAG detection of the receptor in both fractions (Figure 4c,d).

As EPB41L2 expression could affect the total amount of SREB1 protein in the cells through modifications in transcriptional regulation, stability of SREB1 transcripts, or half-life of the protein, we examined the level of SREB1 in total cell extracts after solubilization in Laemmli buffer. No significant change was shown in the total amount of SREB1 in the presence of EPB41L2. Taken together, these results suggest that overexpression of EPB41L2 modifies the membrane environment of SREB1, increasing its solubility when using detergents.

### 3.5. SREB1 Colocalizes with EPB41L2 at the Plasma Membrane of HEK293 Cells

**Figure 5:**
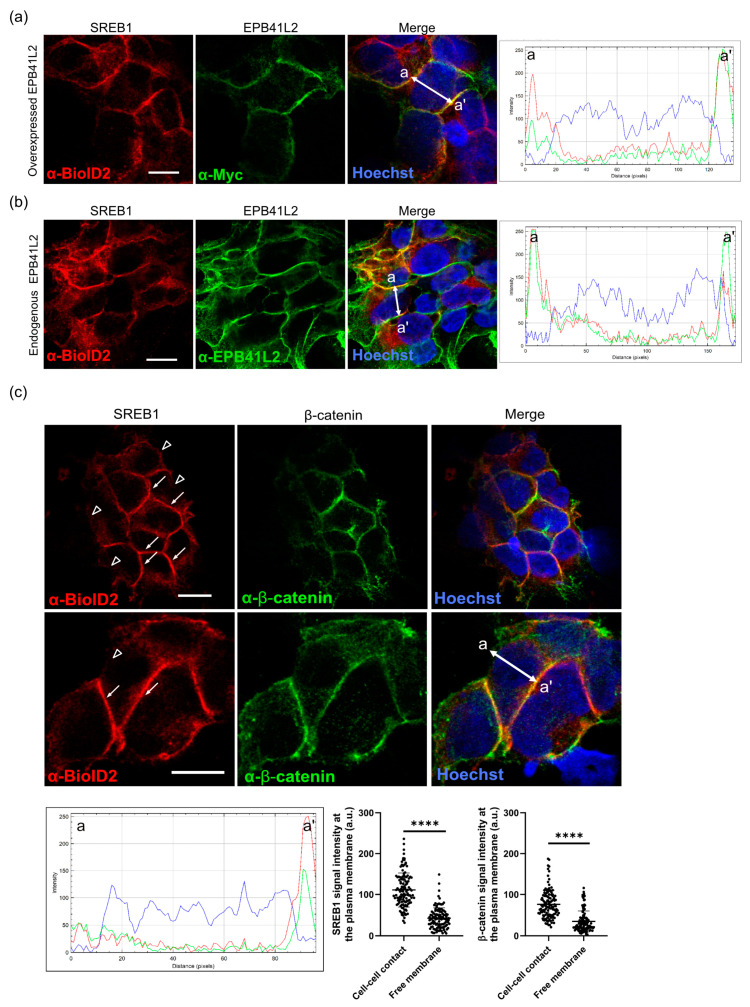
SREB1 colocalizes with EPB41L2 at the plasma membrane and is polarized to cell contacts. Immunofluorescent detection of the SREB1-BioID construct using an anti-BioID2 antibody (red) shows its colocalization at the plasma membrane with overexpressed (**a**) and endogenous (**b**) EPB41L2 (green) detected, respectively, using anti-Myc or anti-EPB41L2 antibodies. RGB plots show the fluorescence intensity profile across one cell (white arrow from a to a’). (**c**) Immunofluorescence staining of β-catenin (green) in cells expressing SREB1-BioID detected using an anti-BioID2 antibody (red) showing more SREB1 in the membranes of cells contacting other cells (arrows) than when in contact with the medium (arrowheads). Nuclei are stained with Hoechst (blue). RGB plots show the fluorescence intensity profile across one cell (white arrow from a to a’). Images were obtained using a confocal inverted Zeiss LSM 780 microscope and a 63× objective. Scale bar: 10 µm. Data are shown as mean intensity value ± SEM for individual membranes. (****) *p*-value < 0.0001.

EPB41L2 is known to form complexes with other proteins near the plasma membrane of the cells. To investigate the intracellular distribution of SREB1 and EPB41L2 and their potential colocalization in the cell, we transiently overexpressed EPB41L2 in HEK293 cells stably expressing SREB1. Immunofluorescence microscopy using anti-BioID2 and anti-Myc antibodies suggested the colocalization of both proteins at the plasma membrane (Figure 5a). Overexpressed SREB1 was also observed in the cytosol. Interestingly, via Western blotting analyses, we found that endogenous EPB41L2 could be detected in untransfected HEK293 cells, using a specific EPB41L2 antibody. Immunofluorescence microscopy using this antibody confirmed the good correlation in the localization of SREB1 and the endogenous form of EPB41L2 (Figure 5b). The images suggested a polarization in the SREB1 localization with a more pronounced signal in membrane domains facing other cells, compared to regions in contact with the medium. To verify this hypothesis, we detected β-catenin, known to be enriched at cell–cell contacts, in cells expressing SREB1. The fluorescence intensity was quantified as 2.6-fold higher in membranes contacting adjacent cells (Figure 5c, arrows) compared to membranes not in contact with other cells (Figure 5c, arrow-heads), suggesting a preferential location of SREB1 at membranes contacting other cells.

### 3.6. EPB41L2 Is Important for Proper Localization of SREB1 to the Plasma Membrane

**Figure 6:**
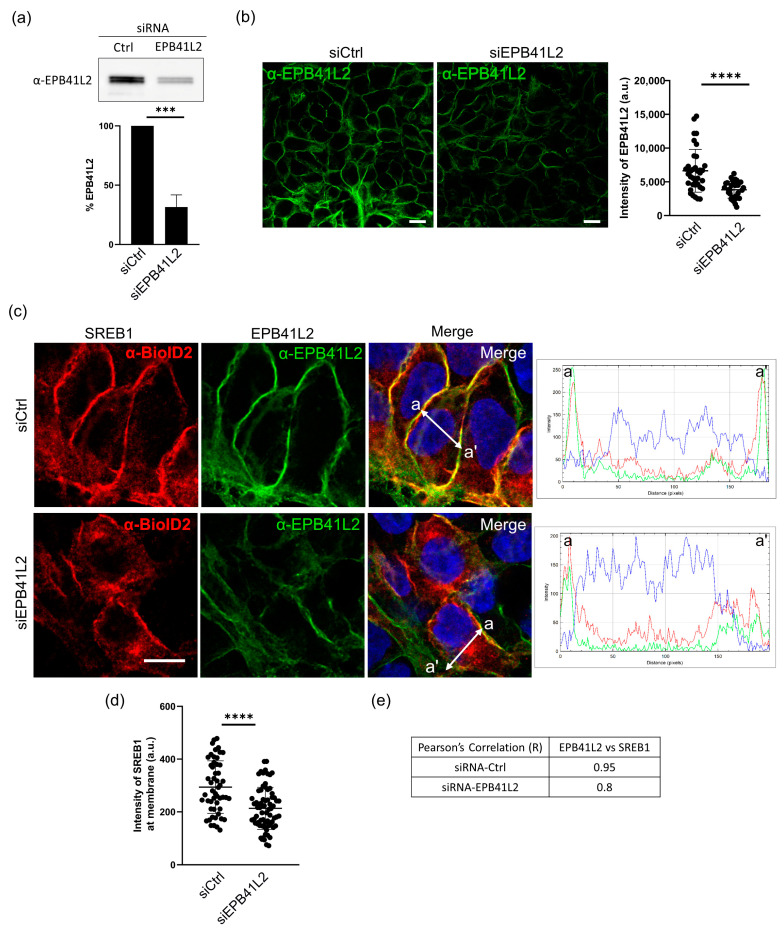
EPB41L2 affects the localization of SREB1 at the plasma membrane. (**a**) Quantification via Western blotting of the percentage of EPB41L2 location at the plasma membrane in HEK293 cells 48 h after transfection of EPB41L2-siRNA or control siRNA (Ctrl). Mean of three independent experiments showing a 68.5% reduction (***) *p*-value < 0.001 (**b**) Confocal immunofluorescence images of EPB41L2 (green) detection in HEK293 cells transfected with control siRNA (siCtrl) or EPB41L2-siRNA (siEPB41L2). Quantification via Image J of the green fluorescence showed a 57.5% decrease. (**c**) Immunofluorescence detection of SREB1-BioD (red) in HEK293 cells transfected with siCtrl or siEPB41L2. Fluorescence intensity plots (white arrow from a to a’) show a reduction in SREB1 (red) expression at the plasma membrane when EPB41L2 (green) is knocked down. Nuclei are stained with Hoechst (blue). Images obtained on a confocal Zeiss LSM 780 microscope with a 63× objective. Scale bar: 10 µm. (**d**) Quantification of the intensity of SREB1 immunofluorescence at the plasma membrane showed a significant decrease of about 1.4-fold in cells transfected with siEPB41L2 as compared to controls. (**e**) Pearson’s correlation coefficient between EPB41L2 and SREB1 fluorescence intensity at the plasma membrane decreased in cells treated with siEPB41L2. Data represent the mean ± SEM of three independent experiments. (****) *p*-value < 0.0001.

Several studies have suggested that EPB41L2 interacts with various GPCRs and contributes to localizing them at the plasma membrane. To examine the impact of the association between SREB1 and EPB41L2 on SREB1 localization in the cell, we investigated whether changes in EPB41L2 expression could influence SREB1 levels at the plasma membrane. Endogenous EPB41L2 was knocked-down by transfection of a cocktail of EPB41L2 siRNA or scrambled non-targeted siRNA (siCTL) as a negative control. We first evaluated the efficiency of EPB41L2 siRNAs using Western blotting and immunofluorescence. We observed that the endogenous expression of EPB41L2 in HEK293 cells decreased by 68.5% in WB and 57.5% in IF, as compared to the siCTL (Figure 6a,b).

To quantify changes in the distribution of SREB1, we measured the fluorescent signal observed at the plasma membrane. Our results showed a decrease of about 1.4-fold in SREB1 levels at the plasma membrane (Figure 6c,d). In these conditions, we also observed a decrease in the correlation coefficient between EPB41L2 and SREB1 localizations. The Pearson’s correlation coefficient decreased from R = 0.95 to R = 0.8 when EP41L2 was knocked down (Figure 6e). Collectively, our results suggest that EP41L2 interacts with SREB1 and plays a significant role in the localization of the receptor at the plasma membrane.

## 4. Discussion

SREB receptors are still considered orphan GPCRs despite different studies suggesting potential endogenous and synthetic ligands [18,19,20,21,22,23,43,44,45]. The lack of validated agonists able to activate SREBs limits the identification of the signaling cascades triggered by these receptors. Classified in the rhodopsin-like family of GPCRs, SREBs lack some of the main molecular determinants considered important for the activation process and the G protein coupling of this family of receptors (i.e., the DRY, CWxP, and NPxxY motifs) [6] and these modified motifs are well-conserved in all vertebrate species including fish [46]. These structural differences may suggest that interactions with protein partners, including G proteins, could be distinct from other GPCR subfamilies. The three SREB present an exceptional conservation of their amino acid sequence in their intracellular loops, particularly the long third loop, compared to their extracellular domains that are somewhat more divergent (Figure S5). These observations suggest that SREBs might share common and original interactors through these highly conserved domains.

To identify potential intracellular partners of SREBs, we took advantage of the proximity-dependent labeling of the promiscuous biotin ligase BioID2 followed by quantitative MS. For this proximity biotinylation assay, we used HEK293 cell lines stably expressing SREB1, SREB2, SREB3, and CMKLR1 C-terminally fused to BioID2. The practical labelling radius of BioID2 is ~10 nm [30]. To increase the biotinylation range of BioID2 beyond that of the ligase alone, we added a 5 nm linker consisting of three repeats of GGGGS between the GPCR and BioID2. As an advantage to prior methodologies, BioID2 is a small ligase that does not affect the localization, expression, or function of the fusion protein. Peak et al. previously investigated the functional response of angiotensin II type 1 and β2-adrenergic receptors to agonists when using a similar approach based on peroxidase-catalyzed proximity-labeling [47], validating the system for GPCRs. However, the longer biotinylation time used in BioID2 assays extends the observation period and enables the identification of partners involved in all steps of the receptor’s lifecycle.

Using this BioID approach, we identified a variety of potential interactors for the SREBs (Figure 2). Although G protein subunits Gαs, Gαi2, and Gα11 were detected at very low abundance as proximal interactors for CMKLR1, they were not identified as such for any of the SREBs. Dupuis et al. reported the absence of ligand-independent G protein coupling of SREB1, suggesting the lack of constitutive activity of this receptor [22]. However, contradicting this, a couple of studies have reported SREB1 constitutive activity, leading to increased inositol phosphate levels, implying that SREB1 couples to the Gq/11 [10] or Gi/o pathways [48]. These discrepancies could be attributed to the different measurement approaches and cell models and the influence of the expression levels of the receptor and reporters in the assays. Similarly, in our assay, none of the β-arrestins were identified as interactors for any of the receptors. In line with this, no β-arrestin 2 recruitment to SREB1 was detected in the absence of ligands in a firefly luciferase assay [22]. Although β-arrestins act as signal transducers in several G protein-independent pathways [49], their primary function involves desensitization and internalization of activated receptors [50]. In our biotinylation approach, we had no evidence of receptor activation, which may explain the absence of arrestin recruitment. Moreover, several receptors such as β3-adrenoceptor [51,52] and the gonadotropin-releasing hormone (GnRH) receptor [53] do not signal through β-arrestins.

The most significant interactors identified for SREBs were SLC3A2, LRBA, and EPB41L2/3 (Figure 2). We confirmed, via co-immunoprecipitations, that these proteins were present in complexes with SREBs and could, therefore, be considered as genuine physical partners (Figure 3).

SLC3A2, also known as the 4F2 heavy chain, is a transmembrane cell-surface protein that forms a heterodimeric complex with one of the light chain proteins known as SLC7. Together, they constitute the system L amino acid transporter, which facilitates the transport of large neutral amino acids such as leucine, phenylalanine, and tryptophan into cells [41]. SLC3A2 was among the most significant interactors of SREB3 but physical interaction with the three SREBs and CMKLR1 was confirmed. Interestingly, the taste GPCRs T1R1/T1R3 were identified as sensors of amino acid availability and were noted to regulate the transport of amino acids in a complex with LAT1 (SLC3A2/SLC7A5), thereby impacting the mammalian target of rapamycin complex 1 (mTORC1) signaling [54]. We searched for SLC7 subunits in our data but none of them were identified in any of the receptor’s interactions. Given that several metabolic functions have been attributed to SREB1, such as being a positive regulator of insulin transcription [10], regulation of glucose homeostasis [8], or involvement in lipid metabolism [11], it would be interesting to further investigate the link between SLC3A2 and SREB1. In the central nervous system (CNS), the SLC3A2/SLC7A11 complex forms the X-C system, the main cysteine/glutamate exchanger that mediates the balance of cystine uptake into neurons and glial cells, playing an important role in cellular oxidative stress regulation [55]. Additionally, amino acid neurotransmitters are controlled by blood–brain barrier solute carrier amino acid transporters, such as LAT1 [56]. A role of SREBs in the regulation of amino acid transport in the CNS can, therefore, be considered.

A second partner, LRBA, was identified as one of the highly enriched candidates interacting with SREB2. LRBA is a ubiquitous cytosolic high-MW protein (319 kDa) reported to be necessary for the vesicular trafficking of the cytotoxic T-lymphocyte antigen 4 (CTLA-4) present in the membrane of activated T cells [57]. Although LRBA is not well studied, 25% of patients with LRBA deficiency are documented to display neurological diseases [58]. Recently, LRBA was proposed as an A-kinase anchoring protein (AKAP) [59]. Similar to other GPCRs [60,61], SREB2 could be part of a complex with an AKAP scaffold protein. We showed that the central region of LRBA, which contains the PH and BEACH domains, was responsible for the interaction with SREB2 (Figure 3d) and CMKLR1 (Figure 3g). Given the extended biotinylation time, LRBA might be involved in the trafficking of SREB2 and CMKLR1 to the plasma membrane, as it is known to be localized in the membrane of intracellular vesicles [62].

EPB41L2 (4.1G) and EPB41L3 (4.1B) were among the most enriched interactors for all SREBs and CMKLR1. While EPB41L2 exhibits ubiquitous expression, EPB41L3 is predominantly expressed in the brain. Both proteins belong to a family characterized by a FERM domain and are known to establish bridges between plasma membrane proteins and the cytoskeleton. We first confirmed that SREB1 and EPB41L2 could be purified as a complex via co-immunoprecipitation (Figure 3a). This interaction was not affected by the presence of 15 µM of the ID5217941 agonist (Figure S4a). The level of EPB41L2 normalized to the level of precipitated SREB1 was measured. No difference in the quantity of immunoprecipitated EPB41L2 was detected indicating no effect of this compound on the interaction of SREB1 with EPB41L2 (Figure S4b). The 4.1 proteins were shown to interact with other GPCRs, including the parathyroid hormone, dopamine D2 and D3, and adenosine receptors, through their third intracellular loop [63,64,65,66]. The 4.1 proteins are also known to interact with the C-terminus of GluR1 [67] and GluR4 [68], thereby affecting their subcellular localization. A trimeric complex composed of 4.1, mGluR1, and SAP97 (a PDZ-domain containing protein) was suggested to promote the synaptic localization of the receptor [67]. In this case a T/SXV PDZ-binding motif at the C-terminus of the receptor was responsible for the interaction with SAP97 [69]. However, no canonical PDZ-binding consensus motif is present at the C-terminus of SREBs, and we did not detect SAP97 among the proximal interactors of SREBs.

Immunofluorescence microscopy performed on HEK293 cells confirmed that SREB1 co-localizes with overexpressed and endogenous EPB41L2 at the plasma membrane. It was previously reported that EPB41L2 colocalized with other GPCRs, PTHR [65] in COS-7 cells, and A1AR in HEK293 cells [64]. Interestingly, SREB1 showed a polarized profile, primarily localizing at β-catenin-associated cell adhesion sites of the plasma membrane (Figure 5). The Wnt/b-catenin pathway plays a major role in neurogenesis, synaptic plasticity, and blood–brain barrier integrity [70,71,72]. Similarly, the presence of SREB1 at cell–cell contacts could imply an essential role in comparable mechanisms, necessitating further investigations. Upon depletion of EPB41L2 by siRNAs, we observed reduced SREB1 levels at the plasma membrane (Figure 6). This protein family has been demonstrated to influence the localization of a wide variety of membrane proteins, including GPCRs, at the plasma membrane [39]. They promote plasma membrane localization of D2 and D3 dopamine [63], adenosine A1a [64], PTH [65], mGluR8a, and mGluR8b receptors [73]. Our findings demonstrate that this phenomenon extends to SREB1. In addition, by performing protein solubilization using different detergents, we observed that EPB41L2 expression increased the solubility of SREB1. It was reported previously that 4.1G interacts with the FcγRI immunoglobulin receptor, impacting its stability in lipid rafts [74]. Ezrin, another FERM domain-containing protein, was identified as a regulator of lipid raft dynamics in B cells [75]. Some GPCRs reside specifically in these well-organized plasma membrane microdomains due to their affinity for the specific lipid composition of lipid rafts [76]. Complexes involving receptors and 4.1 proteins are likely to influence the partitioning of SREBs among membrane microdomains, subsequently modifying the local lipid environment and, in turn, the receptor’s solubility.

In the current study, the identification of binding partners was made in HEK293 cells, which is not optimal as it does not mimic the natural environment of SREBs in neurons. Although the partners identified were reported to be expressed in the brain (https://www.proteinatlas.org/ (accessed on 19 October 2023)), our findings should be validated in more appropriate cellular environments, such as immortalized neurons, to confirm the relevance of these interactions, and potentially uncover new ones.

## 5. Conclusions

In this work, we identified proximal interactors of the orphan SREB receptors as a step toward a better understanding of the intracellular environment and the function of these receptors. We identified EPB41L2, SLC3A2, and domain 3 of LRBA as physical interactors of SREBs. We demonstrated the co-localization of SREB1 and EPB41L2 at the cell surface and the polarized localization of SREB1 in cell–cell contact. Functionally, EPB41L2 was shown to promote the localization of SREB1 in the plasma membrane and to enhance its solubility when using detergents. Further characterization of individual interactors will be necessary to understand the impact of these interactions on the physiological properties of SREBs.

## Data Availability

Raw data are available upon request.

## Supplementary Materials

The following supporting information can be downloaded at: https://www.mdpi.com/article/10.3390/cells12222625/s1, Figure S1: BioID-SREBs are expressed in HEK293 cells; Figure S2: Selection of HEK293 clones stably expressing BioID fusion proteins based on RT-PCR; Figure S3: Pair panel matrices of spectral counts pairwise combinations of all replicates in BioID assay; Figure S4: Physical interactions of SREB1 and SREB3 with EPB41L proteins; Figure S5: Alignment of amino acid sequences of human SREB members; Table S1: Internal primer sequences used to validate the expression of the genes of interest and the housekeeping gene glyceraldehyde phosphate dehydrogenase (GAPDH) (as a reference) in BioID constructs.
