# Supplementary material for: Proximity Interactome Analysis of Super Conserved Receptors Expressed in the Brain Identifies EPB41L2, SLC3A2, and LRBA as Main Partners
Source: Cells. 2023 Nov 14;12(22):2625. doi: 10.3390/cells12222625 (PMC10670248; doi:10.3390/cells12222625)
Supplement: Supplementary file 1 [file cells-12-02625-s001.zip › cells-2653424-supplementary.pdf]

cells

MDPI

Supplementary Materials:

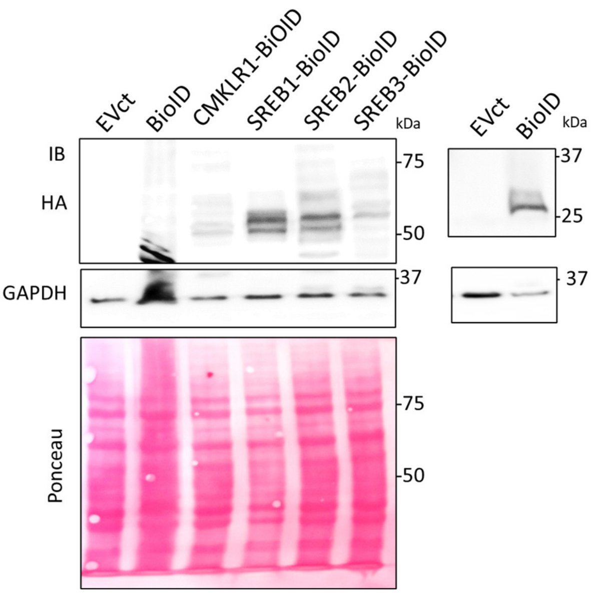

Supplementary Figure S1: BioID-SREBs are expressed in HEK293 cells. Total cell lysates of transfected HEK293T cells were analyzed by Western blotting (following 8% polyacrylamide gels or 12% gels for BioID-alone) and the fusion proteins were detected using anti-HA antibodies (or anti-GAPDH as reference). The protein load was monitored by Ponceau staining. Western blots are representative of three independent experiments.

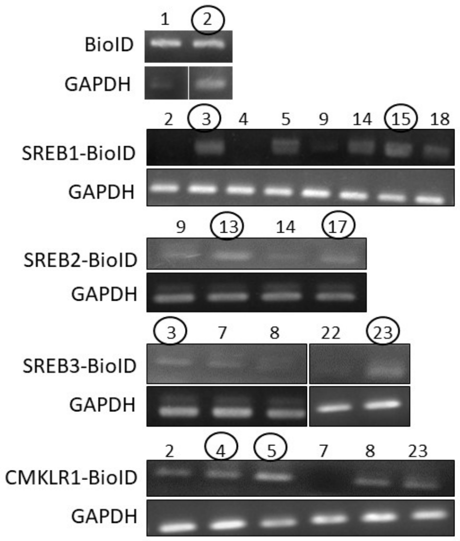

Supplementary Figure S2 : Selection of HEK293 clones stably expressing BioID fusion proteins based on RT-PCR. Clones resulting from G418 selection were analyzed by reverse transcriptase-polymerase chain reaction (RT-PCR) for expression of the receptor-BioID fusions. Representative agarose gels show expression of BioID, SREB1-BioID, SREB2-BioID, SREB3-BioID, and CMKRL1-BioID in different HEK293 clones. The reference gene used was glyceraldehyde phosphate dehydrogenase (GAPDH). Black circles indicate the clones selected for further experiments.

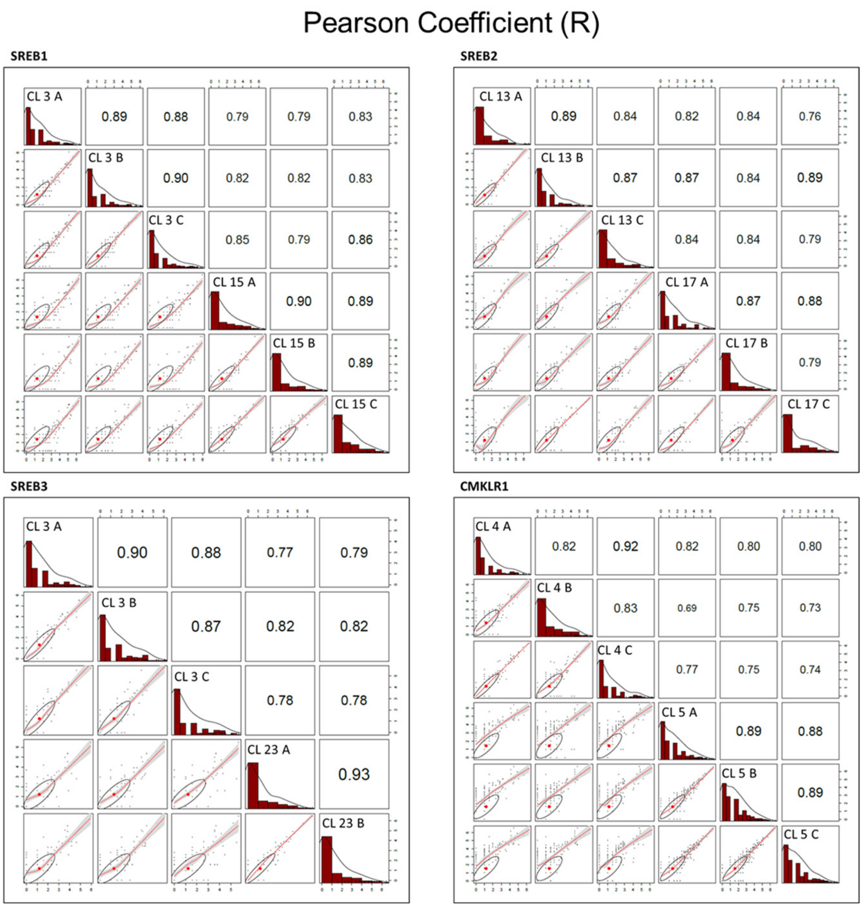

Supplementary Figure S3 : Pair panel matrices of spectral counts pairwise combinations of all replicates in BioID assay. Scatter plots of the number of spectral counts of all combinations of the two biological clones (CL) and three technical replicates (A, B, and C) of each GPCR and its relative Pearson coefficient (R)

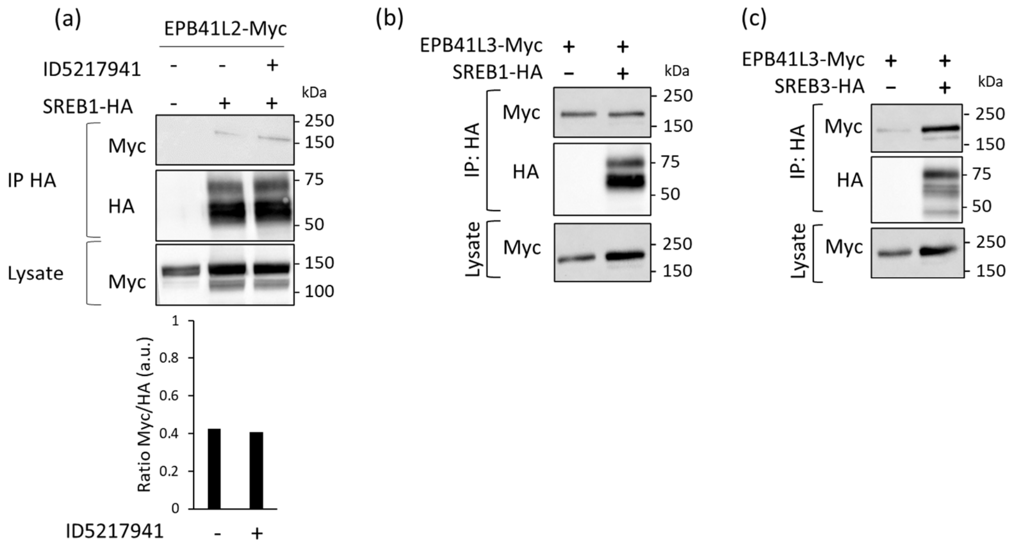

Supplementary Figure S4 : Physical interactions of SREB1 and SREB3 with EPB41L proteins. (a) The interaction of SREB1 and EPB41L2 was not affected by the presence of the ID5217941 agonist. SREB1-BioID2-HA and Myc-tagged EPB41L2 were co-transfected in HEK293T cells and treated with 15 µM of ID5217941. Expression of EPB41L2 was monitored by immunoblotting of the total cell

lysate. Anti-HA immunoprecipitates were analyzed by Western blotting with the indicated antibodies. The EPB41L2-Myc/SREB1-BioID2-HA ratio (quantification using Image J) showed no change in the interaction between SREB1 and EPB41L2 as a result of ID5217941 treatment. HEK293T cells were transfected with (b) SREB1-BioID2-HA or (c) SREB3 -BioID2-HA, together with the proximal interactor EPB41L3 identified in the BioID assay. Expression of the myc-tagged EPB41L3 was confirmed by immunoblotting (Lysate). Anti-HA immunoprecipitates analyzed by Western blotting (b) did not show any specific pull-down of EPB41L3 with SREB1 however (c) EPB41L3 was precipitated with SREB3.

```
                                                                   ICL1
GPR27_HUMAN MANASEPG----GSGGGEAAALGLKLATLSLLLCVSLAGNVLFALLIVRERSLHRAPYYL 56
GPR85_HUMAN MANYSHAADNILQNLSP--LTAFLKLTSLGFIIGVSVVGNLLISILLVKDKTLHRAPYYF 58
GP173_HUMAN MANTTGEPEEVSGALSPPSASAYVKLVLLGLIMCVSLAGNAILSLLVLKERALHKAPYYF 60
            *** :            .     :   :**. *.::: **:.** ::::*:::::**:****:
                                        ECL1
GPR27_HUMAN LLDLCLADGLRALACLPAVMLAARRAAAAAGAPPGALGCKLLAFLAALFCFHAAFLLLGV 116
GPR85_HUMAN LLDLCCSDILRSAICFPFVFNSVKNGS---TWTYGTLTCKVIAFLGVLSCFHTAFMLFCI 115
GP173_HUMAN LLDLCLADGIRSAVCFPFVLASVRHGS---SWTFSALSCKIVAFMAVLFCFHAAFMLFCI 117
            ***** :* :*:  *:* *: :.:..:         .:* **::**:..* ***:**:*: :
                    ICL2                                      ECL2
GPR27_HUMAN GVTRYLAIAHHRFYAERLAGWPCAAMLVCAAWALALAAAFPPVLDGGGD---DEDAPCAL 173
GPR85_HUMAN SVTRYLAIAHHRFYTKRLTFWTCLAV-ICMVWTLSVAMAFPPVLDVGTYSFIREEDQCTF 174
GP173_HUMAN SVTRYMAIAHHRFYAKRMTLWTCAAV-ICMAWTLSVAMAFPPVFDVGTYKFIREEDQCIF 176
            .****:********::*:: * * *: :* .*:*::* *****:* *        *:  * :
                                                          ICL3
GPR27_HUMAN EQRPDGAPGALGFLLLLAVVVGATHLVYLRLLFFIHDRRKMRPARLVPAVSHDWTFHGPG 233
GPR85_HUMAN QHRSFRANDSLGFMLLLALILLATQLVYLKLIFFVHDRRKMKPVQFVAAVSQNWTFHGPG 234
GP173_HUMAN EHRYFKANDTLGFMLMLAVLMAATHAVYGKLLLFEYRHRKMKPVQMVPAISQNWTFHGPG 236
            ::*   *  .:***:*:**::: **: ** :*::* : :***:*.::* *:*::*******

GPR27_HUMAN ATGQAAANWTAGFGRGPTPPALVGIRPAGPGRGARRLLVLEEFKTEKRLCKMFYAVTLLF 293
GPR85_HUMAN ASGQAAANWLAGFGRGPTPPTLLGIRQNANTTGRRRLLVLDEFKMEKRISRMFYIMTFLF 294
GP173_HUMAN ATGQAAANWIAGFGRGPMPPTLLGIRQNGHAA-SRRLLGMDEVKGEKQLGRMFYAITLLF 295
            *:******* ******* **:*:***  .      **** ::*.* **:: :*** :*:**
                      ECL3
GPR27_HUMAN LLLWGPYVVASYLRVLVRPGAVPQAYLTASVWLTFAQAGINPVVCFLFNRELRDCFRAQF 353
GPR85_HUMAN LTLWGPYLVACYWRVFARGPVVPGGFLTAAVWMSFAQAGINPFVCIFSNRELRRCFSTTL 354
GP173_HUMAN LLLWSPYIVACYWRVFVKACAVPHRYLATAVWMSFAQAAVNPIVCFLLNKDLKKCLRTHA 355
            * **.**:**.* **:.:   .**  :*:::**::****.:**.**:: *::*: *: :

GPR27_HUMAN PCCQSPRTTQATHPCDLKGIGL 375
GPR85_HUMAN LYC--RKSRLPREPYCVI---- 370
GP173_HUMAN PCWGTGGAPAPREPYCVM---- 373
                  :    .*  :
```

Supplementary Figure S5: Alignment of amino acid sequences of human SREB members. Putative transmembrane domains are boxed. Relatively conserved GPCR motifs are highlighted in yellow (TRY motif at the junction of TM III and ICL2, NPxxC motif in TM VII, LWxP motif in TM VI). A particularly high amino acid conservation is observed in ICLs of human *GPR27* (SREB1), *GPR85* (SREB2), and *GPR173* (SREB3). Asterisks (*) indicate fully conserved residues, colons (:) strong functional conservation, and dots (.) weak conservation. TM: transmembrane domain, ECL: extracellular loop, ICL: intracellular loop.

Supplementary Table S1: Internal primer sequences used to validate the expression of the genes of interest and the housekeeping gene glyceraldehyde phosphate dehydrogenase (GAPDH) (as a reference) in BioID constructs.

**Gene**	**Sequence**	**Amplicon**	**Position**
SREB1-BioID Forward	TCCTCTTCAACAGGGAGCTG	239 bp	1015-1254 bp
SREB1-BioID Reverse	GTAGCTCACGTTCCACTCCT		
SREB2-BioID Forward	CAGCACAACCCTTCTTTAC	188 bp	1050-1234 bp
SREB2-BioID Reverse	TCACGTTCCACTCCTTCAGTC		
SREB3-BioID Forward	AATTGTCTGCTTCCTGCTCAAC	233 bp	1010-1234 bp
SREB3-BioID Reverse	TCACGTTCCACTCCTTCAGTC		
CMKLR1-BioID Forward	ACTCTTCCTACCCCAGCCATAG	213 bp	1024-1237 bp
CMKLR1-BioID Reverse	TCACGTTCCACTCCTTCAGTC		
BioID-only Forward	CGGGTCTGGAGGCGGGGGTAG	318 bp	12-329 bp
BioID-only Reverse	CACTTCAGGCTGAAGGGGATC		
GAPDH Forward	GGATTTGGTCGTATTGGGCG	240 bp	28-267 bp
GAPDH Reverse	ATCGCCCCACTTGATTTTGG		
